# Investigating Historical Baseflow Characteristics and Variations in the Upper Yellow River Basin, China

**DOI:** 10.3390/ijerph19159267

**Published:** 2022-07-28

**Authors:** Guizhang Zhao, Lingying Kong, Yunliang Li, Yuanzhi Xu, Zhiping Li

**Affiliations:** 1 College of Geosciences and Engineering, North China University of Water Resources and Electric Power, 36 Beihuan Road, Zhengzhou 450045, China; guizhangzhao@163.com (G.Z.); klyyy559@163.com (L.K.); lizhiping@ncwu.edu.cn (Z.L.); 2 Collaborative Innovation Center for Efficient Utilization of Water Resources, 136 East Jinshui Road, Zhengzhou 450046, China; 3 Nanjing Institute of Geography and Limnology, Chinese Academy of Sciences, 73 East Beijing Road, Nanjing 210008, China; 4 Water Resources Research Institute of Shandong Province, 125 Lishan Road, Jinan 250014, China; xuyuanzhixx@163.com

**Keywords:** baseflow estimation, groundwater contribution, Lyne and Hollick digital filter, upper Yellow River, water resource

## Abstract

The baseflow of the Yellow River is vital and important for water resource management and for understanding the hydrological cycle and ecohydrology setting in this arid and semi-arid basin. This study uses a Lyne and Hollick digital filtering technique to investigate the behaviors of the baseflow and the baseflow index in the upper reaches of the Yellow River Basin (China). The observed streamflow discharges along the river were used to analyze the baseflow trend, persistence, and periodic characteristics during the period of 1950–2000. The results show that the average baseflow and BFI in the upper reaches of the Yellow River exhibit a decreasing trend and will continue to decline in the future. Generally, the annual average baseflow and BFI for the most upstream areas of the Yellow River show little difference, while the baseflow and BFI exhibit significant differences for the downstream areas. The filtered annual baseflow varied between 128 × 10^8^ m^3^/year and 193 × 10^8^ m^3^/year for the Yellow River. The BFI ranged from 0.54 to 0.65, with an average of 0.60. This indicates that on average, 60% of the long-term streamflow is likely controlled by groundwater discharge and shallow subsurface flow. Statistics show that two periodic variations were observed in the baseflow evolution process. The results indicate that on average, the first and second main cycles of baseflow behaviors occur at 28 years and 12–17 years, respectively. Correspondingly, the estimation indicates that the abrupt change points tend to appear in the 1960s, the 1980s, and the 1990s. An improved understanding of baseflow behaviors can help guide future strategies to manage the river regime, its water resources, and water quality.

## 1. Introduction

The baseflow refers to the amount of water supplied by groundwater and other delayed water resources to rivers or lakes [1]. It is well-known that the baseflow has been regarded as a main source of surface runoff in the dry season [2,3]. The baseflow plays an important role in affecting hydrological processes and maintaining basic ecological functions of rivers and lakes, guaranteeing industrial and agricultural water, and providing information for conducting non-point-source pollution assessments [4]. Knowledge about the baseflow can improve the understanding of catchment streamflow partitioning, assessing of surface-groundwater flow exchanges, and aiding of water resource management [5,6,7].

The Yellow River Basin is the second largest river basin in China [8]. In recent years, many previous studies have indicated that the surface runoff and groundwater baseflow have decreased significantly due to global climate change and human activities [9,10]. The dynamics and changes in surface water and groundwater resources are most likely to affect municipal, industrial, agricultural, and hydroelectric uses. Additionally, the degraded water resource in this region has seriously restricted the sustainable development of economy and the construction of an ecological environment in northern China [11]. Consequently, it is very important to study the temporal and spatial characteristics of the baseflow and its evolutionary trend in the upper Yellow River over a long period. Understanding baseflow behaviors in the Yellow River is vital for protection and management of groundwater and surface water resources in this region.

The increasingly prominent water resources problems, such as the cut-off of the Yellow River, indicates that more attention should be given to understanding baseflow behaviors. Previous work has demonstrated that the baseflow in the most upstream of the Yellow River (Lanzhou city) accounts for 50% of the water inflow from the upper reaches, but the baseflow tends to decrease every year [12]. Lei et al. [13] investigated the change in baseflow on the Loess Plateau. They found that the baseflow decreased the most in summer and decreased the least in winter. In addition, human activities play a key role in influencing the change in baseflow. Chen et al. [14] estimated the baseflow variations in the source area of the Yellow River. Lei et al. [15] compared the evolution of the baseflow between the windblown sand area and the loess area in a typical tributary of the Yellow River (i.e., the Tuwei River Basin). These previous studies adopted graphical, analytical, and digital filtering techniques to partition the baseflow from the total streamflow. In general, digital filtering methods can remove inconsistencies inherent in graphical methods and reduce the time required for hydrograph separation [16]. Additionally, filtering methods do not have a hydrological basis but aim to generate an objective, repeatable, and easily automated method to obtain baseflow dynamics of a catchment [17]. Thus, digital filtering methods are widely used to generate baseflow components from a wide range of catchment scales.

This previous research regarding the baseflow in the Yellow River focused on either a tributary or a small watershed of the river. That is, these relevant studies are limited in that they do not provide sufficient information regarding the spatiotemporal evolution characteristics of the baseflow in large-scale regions over a long period of time. More knowledge of the baseflow is a first step to understanding how the baseflow may be potentially affected by climate change and/or anthropogenic activities and, in turn, how baseflow changes affect catchment hydrology and the associated ecosystem.

In this study, our specific objectives are to: (1) analyze the temporal and spatial evolution of the baseflow in the upper reaches of the Yellow River from 1951 to 2000 using a digital filtering method and several statistical approaches; (2) explore and discuss the influence of natural factors and human activities on the baseflow and forecast the changing trend of the baseflow in the future.

## 2. Study Area

**Figure 1:**
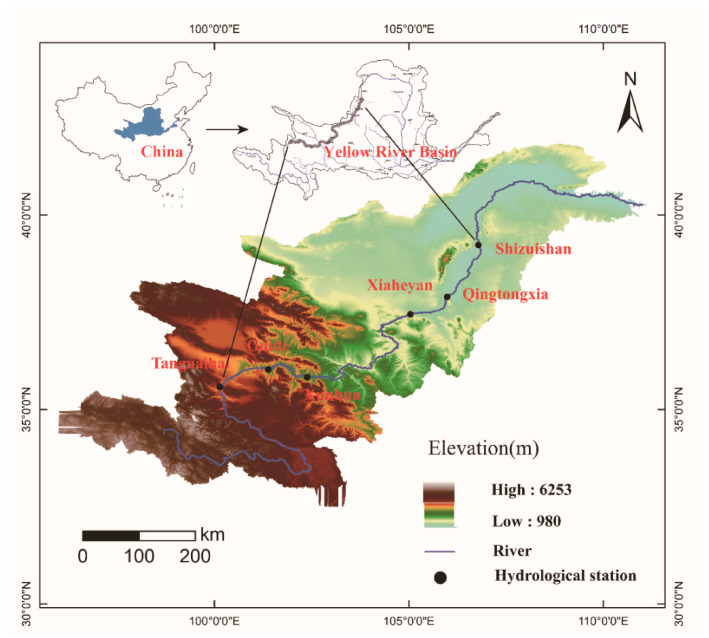
Location of the Upper Yellow River Basin and distribution of hydrological gauging stations.

The Yellow River originates in the Qinghai-Tibet Plateau and follows a sinuous west-to-east route before discharging into the Bohai sea (Figure 1). The upper reaches of the Yellow River stretch from Heyuan Town to Hekou Town, in the Inner Mongolia Autonomous Region [18]. It flows a distance of approximately 3472 km and has a drainage area of 428,000 km^2^ [19]. The terrain of the upper reaches of the Yellow River is complex, with an elevation in the range of 980–6253 m above sea level (Figure 1). The river can be generally divided into three parts from the upstream to the downstream, including the river source, the canyon, and the alluvial plain sections, based on the flow and channel characteristics of the river [20]. The climate condition is mainly a plateau climate and the Upper Yellow River is arid to semi-arid. A 50-year statistic shows that the precipitation of about 406 mm/year is concentrated and unevenly distributed throughout the year, and the daily temperature ranges from 21 °C to 37 °C. The average annual runoff of the Upper Yellow River is 47.73 billion m^3^, accounting for 56% of the whole river [21]. The Upper Yellow River Basin is in the condition of lack of water resources, which is characterized by significant and uneven changes in runoff [22]. Anthropogenic activities have resulted in changes in the hydrological regime in the Upper Yellow River, leading to problems such as water shortages and poor water quality since the 20th century [23].

## 3. Materials and Methods

### 3.1. Data Availability

The daily discharge time series at the six hydrological gauging stations (i.e., Tangnaihai, Guide, Xunhua, Xiaheyan, Qingtongxia, Shizuishan; see Figure 1) were selected to represent the river components for quantifying the baseflow conditions. The daily precipitation and temperature data were also collected and used to analyze the climatic conditions of the Upper Yellow River Basin. All daily hydrometeorological data along the Yellow River are available for the past 50 years and were obtained from the Yellow River Conservancy Commission of the Ministry of Water Resources (Table 1).

### 3.2. Lyne and Hollick Digital Filtering Method

Digital filtering methods are based on signal analysis. The principle is to divide the signal into high-frequency and low-frequency signals, respectively, corresponding to the surface runoff and the baseflow in the runoff [24,25]. This approach was first proposed by Lyne and Hollick [26], and it was further improved and applied in hydrological research [27]. This method is advantageous because it saves time, is highly efficient, has repeatable operations, and has a universal application [28]. Its filtering equation can be given as follows:

(1) $$q_{t}=\alpha q_{t-1}+\frac{\left(1+\alpha \right)}{2}\left(Q_{t}-Q_{t-1}\right)$$

The baseflow equation is:

(2) $$b_{t}=Q_{t}-q_{t}$$

where *q_t_* is the surface runoff at time *t* (m^3^/s); *Q_t_* is the total runoff at time *t* (m^3^/s); *q_t_*_−1_ is the surface runoff at time *t* − 1 (m^3^/s); *Q_t_*_−1_ is the total runoff at time *t* − 1 (m^3^/s); *b_t_* is the baseflow at time *t* (m^3^/s); *α* is the filtering parameters. By comparing and combining multiple cutting effects with the hydrogeological conditions of the basin, a value of 0.95 was selected as the filtering parameter and positive-negative-positive filtering was applied three times [29].

### 3.3. Mann–Kendall Nonparametric Test

The Mann–Kendall test (MK) is a nonparametric statistical test that does not require samples to follow a certain distribution and is not disturbed by a few outliers [30,31]. The statistical variable *S* is defined as:

(3) $$S=\overset{n-1}{\underset{k=1}{\sum }}\overset{n}{\underset{j=k+1}{\sum }}\mathrm{sgn}\;(x_{j}-x_{k})$$

where:

(4) $$\mathrm{sgn}\;\left(x_{j}-x_{k}\right)=\{\begin{array}{c} +1,x_{j}-x_{k}>0\\ 0,\;\;\;x_{j}-x_{k}=0\\ -1,x_{j}-x_{k}<0 \end{array}$$

The standard formula for calculating normal statistical variables is:

(5) $$Z=\{\begin{array}{c} \frac{S-1}{\sqrt{Var\left(S\right)}},S>0\\ 0,S=0\\ \frac{S+1}{\sqrt{Var\left(S\right)}},S<0 \end{array}$$

where Var(S)=n(n−1)(2n+5)/18, a positive *Z* indicates an upward trend, and a negative *Z* indicates a downward trend. When the absolute value of *Z* is greater than or equal to 1.28, 1.64, and 2.32, the distribution indicates that 90%, 95%, and 99% confidence significance tests have been passed [32].

When the MK test is further used to detect sequence mutations, the test statistic is different from the *Z* above. By constructing an order column [33]:

(6) $$S_{k}=\overset{k}{\underset{i=1}{\sum }}\overset{i-1}{\underset{j}{\sum }}a_{ij}\;\;(k=2,3,4\cdots ,n)$$

where:

(7) $$a_{ij}=\{\begin{array}{c} 1,x_{i}>x_{j}\\ 0,x_{i}<x_{j} \end{array}\;\;\;\;\;1\;\leq \;j\leq \;i$$

Define a statistical variable:

(8) $$UF_{k}=\frac{\left[S_{k}-E\left(S_{k}\right)\right]}{\sqrt{Var\left(S_{k}\right)}}\;\;(k=1,2,\cdots ,n)$$

where *E*(*S_k_*) is the mean of *S_k_*; *Var*(*S_k_*) is the variance of *S_k_*.

The inverse order of the time series *x_n_*, *x_n_*_−1_,…, *x*_1_, repeats this process to obtain the statistical variables *UB_k_* (*k* = *n, n* − 1,…, 1). At the same time: *UF_k_* = −*UB_k_*. When the two curves of *UB_k_* and *UF_k_* intersect, and the *U* value at this point satisfies <1.96, then it is considered as the mutation point of the sequence. The test confidence level is equal to 0.05.

### 3.4. Rescaled Range Analysis Method

The Rescaled Range analysis method (*R*/*S*) was proposed and further developed as a fractal theory to analyze time series data [34,35]. This method can determine whether the future change characteristics of time series data are consistent with the past. Recently, the R/S analysis has been widely used in hydrology and earth sciences. The main principle is that the time series {ζ (*t*)}, *t* = 1, 2, …… for any positive integer *τ* ≥ 1,

Define mean series:

(9) $$<\xi {>}_{\tau }=\frac{1}{\tau }\overset{\tau }{\underset{t=1}{\sum }}\xi \left(t\right),\;\;\tau =1,\;2,\cdots ,$$

Cumulative deviation:

(10) $$\;X\left(t,\tau \right)=\overset{\tau }{\underset{t=1}{\sum }}(\xi \left(t\right)-<\xi {>}_{\tau }),\;\;\;1\leq t\leq \tau$$

Range:

(11) $$R\left(\tau \right)=\underset{1\leq t\leq \tau }{\max}X\left(t,\tau \right)-\underset{1\leq t\leq \tau }{\min}X\left(t,\tau \right)\;,\;\;\;\tau =1,\;2,\cdots ,$$

Standard deviation:

(12) $$S\left(\tau \right)=\sqrt{\frac{1}{\tau }\overset{\tau }{\underset{t=1}{\sum }}\left(\xi \left(t\right)-{<\xi >}_{\tau }{)}^{2}\right)}\;\;\;\tau =1,\;2,\cdots ,$$

Define *R*(τ)/*S*(τ) = *R*/*S*. If *R*/*S*∝*τ*, *H* exists. It shows that the Hurst phenomenon exists in the time series analyzed. *H* is called the Hurst index. This value can be obtained by the least-squares fitting formula in the dual coordinate system according to the calculated *τ* value and *R*/*S* value [35]. When 0 < *H* < 0.5, it indicates that the time series has a long-term correlation, but the future development trend is opposite to the past. When *H* = 0.5, it indicates that the time series are mutually independent and completely random, and the future development trend has nothing to do with historical data. When *H* > 0.5, it indicates that the time series has a long-term correlation, and the future development trend is consistent with past changes. The closer *H* is to 1, the more persistent it is.

### 3.5. Wavelet Analysis

Wavelet analysis is a local analysis of time and frequency and it performs a multi-scale analysis of signals or functions through the operation of stretching and translation [36]. Eventually, a time subdivision at high frequency and frequency subdivision at low frequency are achieved. This method is one of the greatest breakthroughs in the scientific method since the Fourier transform. In recent years, the wavelet method has been used widely in meteorology, hydrology, and other fields, such as hydrological series prediction and hydrological series periodicity analysis [37].

The specific principle is expressed as follows:

(13) $$W_{f}\left(a,b\right)={\left|a\right|}^{-1/2}\int_{-\infty }^{\infty }f\left(t\right)\times \phi \left(\frac{t-b}{a}\right)db$$

(14) $$V\left(a\right)=\int_{-\infty }^{\infty }{\left|W_{f}\left(a,b\right)\right|}^{2}db$$

where *W_f_*(*a*, *b*) is the wavelet transform coefficient; *f*(*t*) is the hydrological time series; *Φ*(*t*) is the wavelet function; *a* is the scale factor; *b* is the time factor; *V*(*a*) is the wavelet variance.

## 4. Results and Discussion

### 4.1. Spatiotemporal Characteristics of Baseflow and BFI

The Lyne and Hollick digital filtering method was used to analyze the variation characteristics of the baseflow at six hydrological stations in the upper reaches of the Yellow River, as shown in Table 2. The annual average baseflow of the Heyuan section (e.g., the Tangnaihai and Guide stations) does not exhibit large differences, with the baseflow volume in the range of 127 × 10^8^–129 × 10^8^ m^3^/year. The annual average baseflow of the canyon section (e.g., the Xunhua, Xiaheyan, and Qingtongxia stations) varies greatly, with a variation by up to around 40%. The maximum annual average baseflow is observed at the Xiaheyan gauging station (i.e., up to 193.38 × 10^8^ m^3^/year), while the minimum annual average baseflow can be found at the Tangnaihai station (i.e., ~127.76 × 10^8^ m^3^/year). Additionally, the baseflow for the upper reaches of the Yellow River differs greatly between years. The annual variance of the Qingtongxia station is higher than those of other stations (see Table 2). That is, the variation coefficient (*C_v_*) and extreme ratio (*K*) of the annual baseflow for this station are 0.38 and 5.22, respectively.

**Figure 2:**
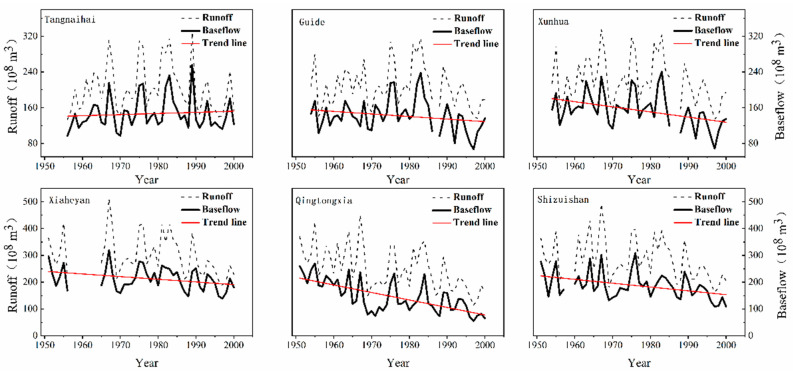
Interannual variation of river discharge and baseflow volume.

The trends of the daily runoff series and associated baseflow at the hydrological stations were investigated using the MK test method, as shown in Figure 2. The results show that, in general, the baseflow in the upper reaches of the Yellow River exhibits a distinct decline trend. For example, the *Z*-value of the annual baseflow of the Guide Station is 8, which passes the significance test of 90% confidence, and the downward trend is not obvious. The descending trend in the baseflow of the Qingtongxia station is the most significant, with a *Z*-value of 4.23, which passes the significance test of 99% confidence. From 1951 to 2000, the precipitation in the upper reaches of the Yellow River decreased, which reduced the supply of the baseflow, and the temperature increased significantly [38,39], which may increase the consumption of the baseflow. It can be accepted that the baseflow is not only affected by climate change, but also affected by human activities. The reservoir storage and the increase in industrial and agricultural water consumption are the main anthropogenic reasons for the decrease in baseflow in the upper reaches of the Yellow River [40,41]. Additionally, increasing animal husbandry and mining activities damaged the original underlying surface, significantly reducing groundwater resource conservation function, and thereby reducing the baseflow [42]. Increasing urbanization and industrialization in the upper reaches of the Yellow River and the construction of the “Three North” Shelterbelt System are most likely to increase the area of woodland and cultivated land as well as the associated groundwater exploitation [43,44], potentially leading to a decreasing trend in the baseflow.

**Figure 3:**
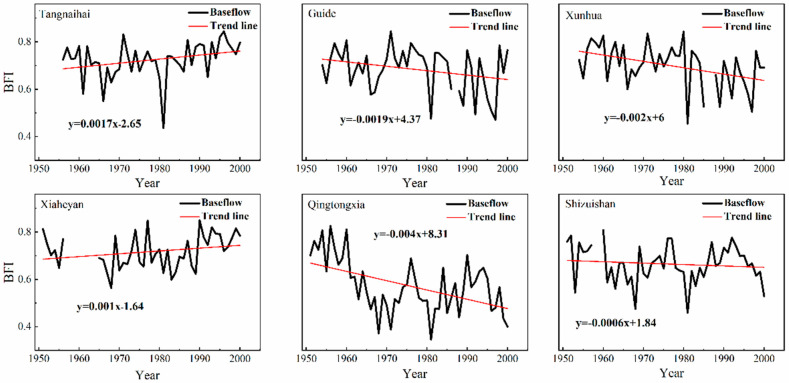
Interannual variation in BFI at all the hydrological gauging stations.

The baseflow index (BFI) is defined as the ratio of baseflow to total runoff. It can reflect the recharge characteristics of groundwater to rivers. As shown in Figure 3, the proportion of the baseflow to the runoff in the upper reaches of the Yellow River is relatively large, and the BFI shows a downward trend on the whole, with a distinct annual variation. The Qingtongxia hydrological station exhibits the most significant downward BFI trend. In addition, the decline rate of the annual variation at this station can be up to approximately 0.4%, while BFI increased slightly at Tangnaihai and Xiaheyan stations (Figure 3).

**Figure 4:**
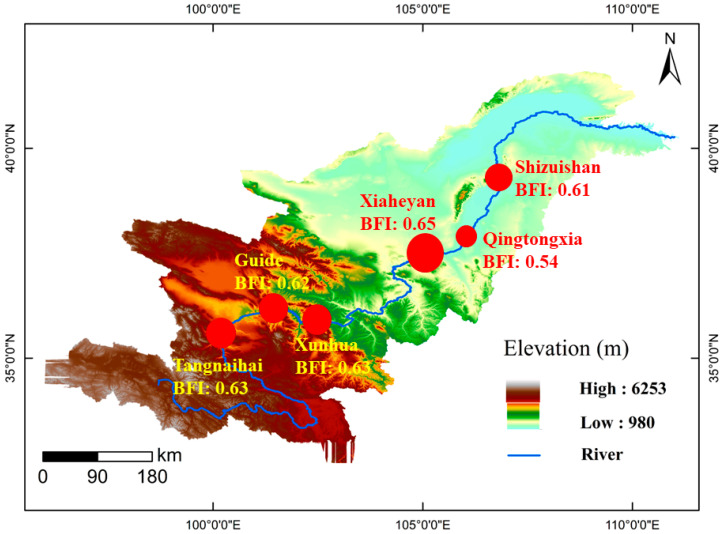
Spatial distribution in the calculated BFI in the upper reaches of the Yellow River. The bigger the circle, the larger its value.

From the headwaters of the upper reaches of the Yellow River to the alluvial plain, the average annual BFI decreases first and then increases (Figure 4). From Tangnaihai station to Xiaheyan station, the annual average BFI change trend is not obvious, ranging from 0.62 to 0.65. The average BFI of the Qingtongxia station decreased significantly and reached a minimum value of 0.54, and the average BFI of the Shizuishan station increased slightly. The Qingtongxia station is located on the Ningxia plain where the irrigation and agriculture often occur [38]. While diverting water from the Yellow River for irrigation, a large amount of groundwater is also exploited for irrigation, resulting in a reduction in baseflow contribution [45]. In addition, the construction of the Qingtongxia water control project has raised the base level of groundwater discharge, thus causing a possible decreasing BFI trend. For the Shizuishan station, part of the recession water in the irrigation areas returns to the Yellow River along the Shizuishan River, and the other part returns to the groundwater system through irrigation infiltration [46]. The decreasing of groundwater exploitation is likely to increase the groundwater BFI.

### 4.2. Abrupt Change in Baseflow

**Figure 5:**
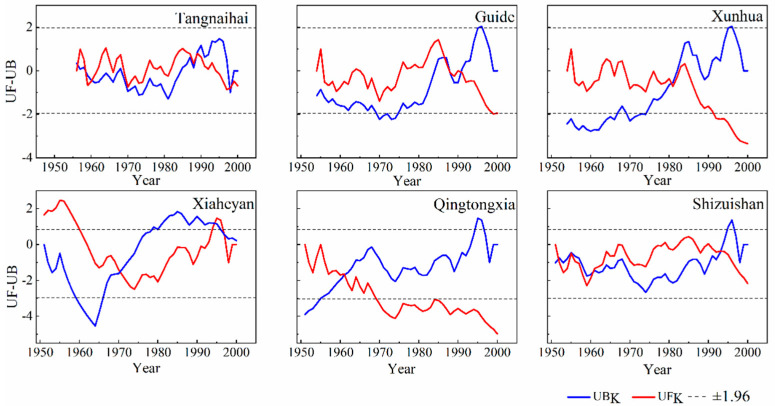
MK trend analysis for the baseflow at the six hydrological gauging stations.

The baseflow of the upper reaches of the Yellow River changed abruptly during the period of 1950s–1990s (Figure 5). The changes in the River section of the Qingtongxia and the Shizuishan occurred in the 1960s, and the section of the Guide and the Xunhua occurred in the 1980s. The impoundment of the Qingtongxia and the Longyangxia reservoirs should be responsible for the abrupt change in the baseflow. In general, there are two major reasons for the abrupt change in the baseflow of the six stations in the 1990s. First, the rainfall in the upper reaches of the Yellow River has decreased significantly since the 1980s, and the baseflow in the Yellow River Basin mainly depends upon the rainfall. Second, various human activities (e.g., reservoir construction and groundwater exploitation) may affect the vegetation pattern and the associated land surface conditions, thus weakening the contribution of rainfall to the baseflow behavior.

### 4.3. Periodic Characteristics and Trend Assessment of Baseflow

**Figure 6:**
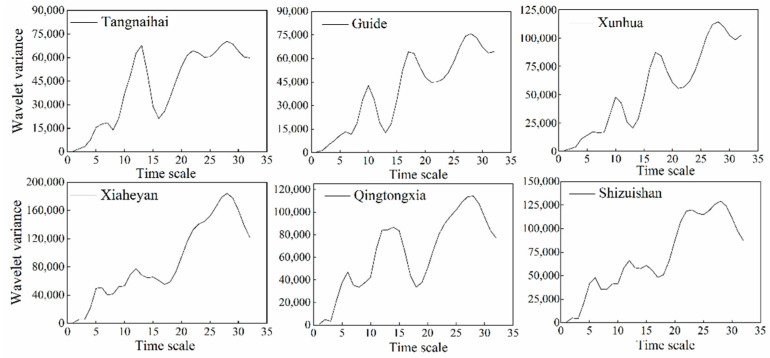
Wavelet variance diagram of the baseflow time series at the six hydrological stations.

**Figure 7:**
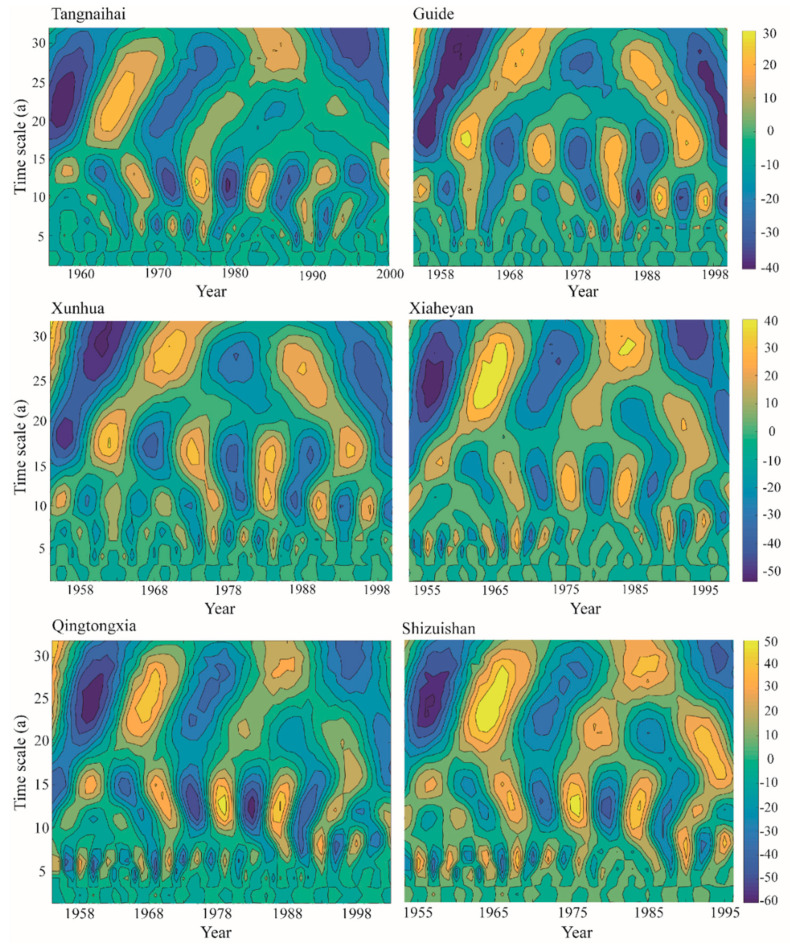
Wavelet analysis of the baseflow time series at the six hydrological stations.

The evolution of baseflow is characterized by multiple time scales in the upper reaches of the Yellow River. The contour map of the wavelet analysis can reflect the periodic change in baseflow sequences across multiple time scales. When the wavelet coefficient is positive, it means that the baseflow experiences a wet period; when the wavelet coefficient is negative, it means that the baseflow experiences a dry period. The wavelet variance diagram reflects the periodic fluctuation and energy of the baseflow sequence at various time scales, and its peak position is the main time period of the baseflow sequence. From the wavelet variance diagram (see Figure 6), it can be found that the six hydrological stations have two obvious peaks. The first peak is at the time scale of 28 years, which is the first main cycle of baseflow change, showing two obvious alternating wet and dry periods. The second peak is at the time scale of about 12–17 years, characterized by five alternating wet and dry periods. The wavelet results further show that, on average, the time scale of the baseflow in the Upper Yellow River can occur with periods from approximately 10 days to 28 days, as illustrated by Figure 7. This finding is consistent with the periodic variation in precipitation in the upper reaches of the Yellow River. Generally, the fluctuation of the two periods controls the variation characteristics of the baseflow in the upper reaches of the Yellow River over the total time domain.

**Figure 8:**
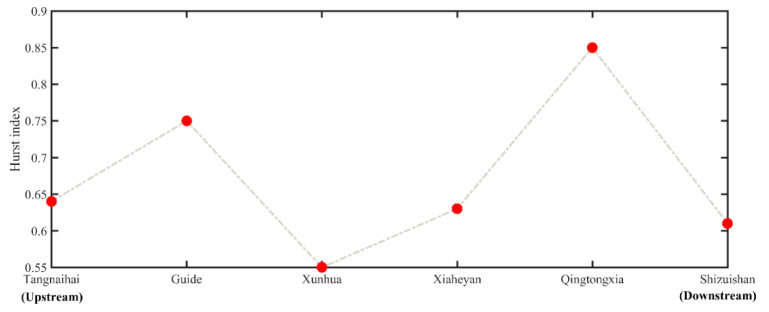
Hurst index for the baseflow trend forecast at the six hydrological stations.

Figure 8 further shows the trend forecast using the Hurst index. The Hurst index of the six hydrological stations in the upper reaches of the Yellow River is greater than 0.5. This result (i.e., *H* > 0.5) indicates that there is a long-term positive correlation between the future and the past 50 years (see Section 3.4). Therefore, the baseflow of the upper reaches of the Yellow River is likely to exhibit a decline trend.

## 5. Conclusions

The baseflow in rivers has been shown previously to have important implications for the river’s ecosystem and water quality. The current study was based on observed datasets to interrogate the spatiotemporal characteristics in baseflow behaviors and the influence of natural factors and human activities on the baseflow in the Upper Yellow River (China). The baseflow conditions and its causal factors were investigated using the digital filtering approach and statistical methods.

The results indicate that the baseflow and the BFI of the upper reaches of the Yellow River generally show a downward trend. Spatially, the annual average baseflow and BFI for the most upstream areas of the Yellow River show little difference, while the baseflow and BFI exhibit significant differences for the downstream areas of the river. Large changes are observed for the annual average baseflow from the canyon section to the alluvial plain, with a maximum variation by up to around 40%. The MK test shows that the baseflow of the upper reaches of the Yellow River exhibits abrupt changes during the study period, particularly in the 1960s and the 1980s. Wavelet analysis indicates two obvious alternating wet and dry periods. In addition, the first main cycle for the baseflow series is around 28 years, and the second main period is between 12 and 17 years. The R/S analysis demonstrates that the baseflow in the Upper Yellow River tends to exhibit a decline trend in future. Rainfall and human activities (e.g., reservoir construction and groundwater exploitation) may play an important role in influencing baseflow behaviors. These findings provide useful information for the future river management and other similar river systems worldwide. Further work for managers and ecologists studying the Yellow River should consider the contribution of the baseflow to the quality and ecosystem of the river, as 60% of the streamflow comes from the baseflow through subsurface flow paths. Additionally, the next study will focus on the contribution of various factors to the spatial variation in the baseflow, particularly the anthropogenic influence since 2000.

## Figures and Tables

**Table 1:** Basic information of the hydrological gauging stations.

Station	Geographical Location	Drainage Area (km^2^)	Series Length
Tangnaihai	100°08′ E, 35°30′ N	121,972	1956–2000
Guide	101°23′ E, 36°02′ N	133,650	1954–2000
Xunhua	102°23′ E, 35°50′ N	145,459	1954–2000
Xiaheyan	105°02′ E, 37°27′ N	254,142	1951–2000
Qingtongxia	105°59′ E, 37°54′ N	275,010	1951–2000
Shizuishan	106°47′ E, 39°14′ N	209,146	1951–2000

**Table 2:** Statistics of baseflow characteristics in the study area.

Station	Coefficient of Variation	Annual Baseflow (10^8^ m^3^/year)			Extreme Ratio
	*C_v_*	Max.	Min.	Ave.	*K*
Tangnaihai	0.23	213.76	90.66	127.76	2.36
Guide	0.22	199.87	67.10	129.80	2.98
Xunhua	0.20	204.57	83.44	141.88	2.45
Xiaheyan	0.20	296.48	128.07	193.38	2.31
Qingtongxia	0.38	274.12	52.47	137.2	5.22
Shizuishan	0.23	258.80	95.31	170.21	2.71

## Data Availability

The data presented in this study are available on request from the corresponding author.
